# A Method of Water COD Retrieval Based on 1D CNN and 2D Gabor Transform for Absorption–Fluorescence Spectra

**DOI:** 10.3390/mi14061128

**Published:** 2023-05-27

**Authors:** Meng Xia, Ruifang Yang, Nanjing Zhao, Xiaowei Chen, Ming Dong, Jingsong Chen

**Affiliations:** 1 Key Laboratory of Environmental Optics and Technology, Anhui Institute of Optics and Fine Mechanics, Chinese Academy of Sciences, Hefei 230031, China; mxia@aiofm.ac.cn (M.X.); rfyang@aiofm.ac.cn (R.Y.); xwchen@aiofm.ac.cn (X.C.); mdong@aiofm.ac.cn (M.D.); cjs1998@mail.ustc.edu.cn (J.C.); 2 Science Island Branch of Graduate School, University of Science and Technology of China, Hefei 230026, China; 3 Institutes of Physical Science and Information Technology, Anhui University, Hefei 230601, China

**Keywords:** UV-vis absorption spectrum, 3D fluorescence spectrum, COD, 2D Gabor transform, spectral feature fusion, convolutional neural network

## Abstract

Chemical Oxygen Demand (COD) is one of the indicators of organic pollution in water bodies. The rapid and accurate detection of COD is of great significance to environmental protection. To address the problem of COD retrieval errors in the absorption spectrum method for fluorescent organic matter solutions, a rapid synchronous COD retrieval method for the absorption–fluorescence spectrum is proposed. Based on a one-dimensional convolutional neural network and 2D Gabor transform, an absorption–fluorescence spectrum fusion neural network algorithm is developed to improve the accuracy of water COD retrieval. Results show that the RRMSEP of the absorption–fluorescence COD retrieval method is 0.32% in amino acid aqueous solution, which is 84% lower than that of the single absorption spectrum method. The accuracy of COD retrieval is 98%, which is 15.3% higher than that of the single absorption spectrum method. The test results on the actual sampled water spectral dataset demonstrate that the fusion network outperformed the absorption spectrum CNN network in measuring COD accuracy, with the RRMSEP improving from 5.09% to 1.15%.

## 1. Introduction

In recent years, water pollution has become one of the major environmental problems that affect sustainable development worldwide. Water pollution not only harms human health but also disrupts the balance of ecosystems, hindering sustainable development. Organic pollution is the main type of water pollution in surface water. Severe organic pollution can lead to a rapid decrease in dissolved oxygen in water, posing a threat to the health of aquatic organisms and disrupting the balance and stability of aquatic ecosystems [1,2]. In addition, organic pollution of water bodies can cause odors, discoloration, foaming, and toxic small-molecule organic compounds. Long-term exposure to these compounds can increase the risk of cancer and teratogenicity [3]. Therefore, it is necessary to use scientific and effective indicators to evaluate organic pollution in water bodies and to monitor it effectively. Chemical oxygen demand (COD) is an important indicator used to measure organic pollution in water bodies. It is defined as the amount of oxygen consumed by the oxidation and decomposition of organic matter under controlled reaction temperature and time conditions [4]. Rapid and accurate detection of COD is of great significance for routine water quality monitoring, sewage treatment, and pollution source monitoring [5].

Currently, the ultraviolet-visible (UV-Vis) absorption full-spectrum method is the most widely used method for water quality detection. The large amount of data and multiple characteristic information contained in full-spectrum analysis is conducive to the development of spectral analysis retrieval algorithms, which grants the UV-Vis absorption full-spectrum method great potential for application and research value. The UV-Vis absorption spectrum method also has advantages such as fast analysis speed and low cost, making it widely used in rapid analysis of cross-sectional water quality and real-time environmental monitoring [6,7,8]. However, in some cases, the UV-Vis absorption spectrum method may not offer sufficient specificity or sensitivity. In comparison, fluorescence spectroscopy has many unique advantages, such as high specificity, sensitivity, and detection rates, which can accurately distinguish target fluorescent molecules from other molecules and avoid misjudgment. These features make it widely used for detecting organic pollutants in water bodies [9,10].

In terms of fluorescence spectral data analysis methods, with the development of computer technology, more and more new data feature extraction methods have been applied to three-dimensional fluorescence spectra. The traditional classical algorithms for feature extraction from fluorescence spectra are dimensionality reduction methods represented by the PCA method [11] and the PARAFAC method [12]. However, fluorescence spectral data are high-dimensional data, and using these dimensionality reduction methods for feature extraction from fluorescence spectra will inevitably lead to information loss. In addition, these algorithms typically cannot handle nonlinear data well, while three-dimensional fluorescence spectral data typically has complex nonlinear relationships, leading to large errors when using traditional dimensionality reduction methods for data retrieval. Therefore, there has been research to transfer deep learning algorithms to fluorescence spectral feature extraction, in order to learn and extract complex high-dimensional nonlinear components in three-dimensional fluorescence spectra [13,14,15] Although these methods have achieved high accuracy in identifying three-dimensional fluorescence spectra after successful model training, there are still many issues when deploying convolutional neural networks for training and modeling fluorescence spectral data.

Firstly, due to the limitations of fluorescence spectrometer instruments, the amount of fluorescence spectral data is relatively small compared to the amount of image data or traditional tabular data, which is not conducive to accurate neural network training. Secondly, the high dimensionality and sparsity of fluorescence spectral data itself also increase the difficulty of data processing. These data characteristics of fluorescence spectra can lead to the problem of small sample size during neural network model training, which can cause overfitting of the model and affect its generalization performance [16]. Additionally, fluorescence spectra may have an internal filtering effect (IFE) [17] during measurement, which refers to the overlap between absorption and fluorescence emission due to the interaction between fluorescence molecules in high-concentration fluorescence samples. Therefore, in high-concentration fluorescence samples, the position and shape of the fluorescence signal may be distorted due to IFE [18], which can lead to an increase in error when using interpolation methods for data augmentation and make it difficult to expand the fluorescence dataset. Therefore, traditional CNN models often cannot fully exploit the features of fluorescence spectra.

Compared with traditional dimensionality reduction algorithms and CNN models, image feature extraction algorithms in the field of digital image processing, such as Gabor transform [19] Hotelling transform [20], and maximum stable extremal region method [21], are more suitable for extracting graphical information from fluorescence spectral data. The advantages of these algorithms are: (1) These image feature extraction algorithms usually have clear physical or geometric meanings in the calculation process, so they have good interpretability for the extracted fluorescence spectral features. (2) These algorithms usually have a certain parameter adjustment space, which can be adjusted and optimized according to the actual problem, and can also adapt to different fluorescence spectral datasets and application scenarios, with good scalability. (3) From the perspective of the small amount of fluorescence spectral data, using image feature extraction algorithms in the field of digital image processing has great advantages. These algorithms can usually handle high dimensionality and sparsity problems and can extract key feature information through specific processing methods of fluorescence spectral data. At the same time, these algorithms have been widely used in the field of images, and their application and improvement methods are more mature, providing more reliable and effective methods for fluorescence spectral feature extraction. For example, Huang et al. [22] proposed a method using two-dimensional Gabor wavelet transform to extract features from the fluorescence spectra of various organic mixtures and used support vector machines to classify the solutions. The results showed that the two-dimensional Gabor wavelet transform is more effective than the PCA method for feature extraction of three-dimensional fluorescence spectra.

In the detection of organic pollutants in water bodies, the use of three-dimensional fluorescence spectroscopy technology can effectively extract information on organic matter in water. However, due to IFE, the fluorescence spectra of high-concentration samples may be distorted, which may mislead feature extraction algorithms and results in incorrect identification of the components of organic matter. In order to solve the impact of internal filter effects on fluorescence signals, the most common method is to measure the absorption spectrum of the sample at the same time and use the combined absorption spectrum peak position and intensity to correct the IFE [23]. Additionally, it is necessary to consider that different organic components contribute different amounts to COD, and the absorption spectrum peaks and fluorescence spectrum peaks of different organic components may overlap. By fusing spectral information of absorption and fluorescence, the diversity and amount of information in the data can be increased, thereby improving the accuracy and sensitivity of detecting organic pollutants in water bodies. Therefore, spectral fusion methods have broad application prospects in the detection of organic pollutants in water bodies.

Currently, common information fusion methods include weighted averaging, linear combination, ensemble learning, Bayesian network, D-S evidence theory, fuzzy logic, set theory, Kalman filtering, and other methods [24]. From the perspective of information fusion hierarchy, these methods can be divided into four categories from low to high levels: data-level fusion, feature-level fusion, decision-level fusion, and task-level fusion [25]. From the perspective of fusion hierarchy, the fusion level between the water sample absorption spectrum and the three-dimensional fluorescence spectrum is mainly feature-level fusion [26]. This is determined by the data characteristics of these two spectral data: (1) The dimensions between spectral data are imbalanced. The absorption spectrum is comprised of one-dimensional data, while three-dimensional fluorescence spectrum is comprised of two-dimensional image data. The imbalanced dimensions between the two datasets may cause the fluorescence spectrum data to obscure the absorption spectrum data when directly fused. Therefore, it is usually necessary to use dimensionality reduction on fluorescence spectrum data before fusing them with absorption spectrum data. (2) The dataset sizes of different spectral data are imbalanced. Due to differences in spectral acquisition speed, absorption spectrum acquisition is often faster, resulting in a larger dataset size. On the other hand, the acquisition speed of the three-dimensional fluorescence spectrum is slower, resulting in a smaller dataset size. The imbalanced dataset size may cause the classifier to lean towards learning the larger class, thereby affecting the accuracy of information fusion.

Dealing with imbalanced datasets can typically be addressed using feature learning methods, where the original data are transformed or reduced to a new feature space that makes it easier to distinguish between different datasets [27,28]. Alternatively, ensemble learning methods can be used to combine multiple individual classifiers into a strong classifier, improving the classification performance [29,30]. These methods have also been applied in spectral data fusion. For instance, Hu et al. [31] proposed a feature extraction method for fast analysis of water pollutants based on three-dimensional fluorescence and absorption spectra. They fused fluorescence spectral features with absorption spectral features by using Stokes Shift to greatly improve the speed of spectral feature extraction. Guan et al. [32] proposed a COD online detection method for water quality based on multi-source spectral feature hierarchical fusion using Discrete Wavelet Transform (DWT) and Stepwise Projection Algorithm (SPA) to denoise and extract features from two types of spectral data and a weighted averaging method to fuse the two feature vectors for modeling. Compared to a single spectral model, the fused spectral method reduced the mean squared error of COD prediction by about 60%.

This paper focuses on improving the speed and accuracy of COD detection in water using absorption and fluorescence spectra. Specifically, the paper addresses the issue of low accuracy of COD retrieval using a single absorption spectrum, as well as the problem of dimension imbalance in the fusion analysis of absorption and fluorescence spectra. A COD retrieval algorithm based on absorption–fluorescence spectral feature fusion is proposed. The one-dimensional convolutional neural network algorithm is used to extract the UV-visible absorption spectral features of water COD, and the 2D Gabor transform algorithm is used to extract the three-dimensional fluorescence spectral features of water COD. A neural network algorithm for absorption–fluorescence spectral feature data fusion is developed to achieve rapid COD retrieval based on feature fusion of absorption and fluorescence spectra, thereby addressing the problem of large COD retrieval errors in fluorescence organic matter using a single spectrum method.

## 2. Materials and Methods

### 2.1. Hardware Setup

**Figure 1:**
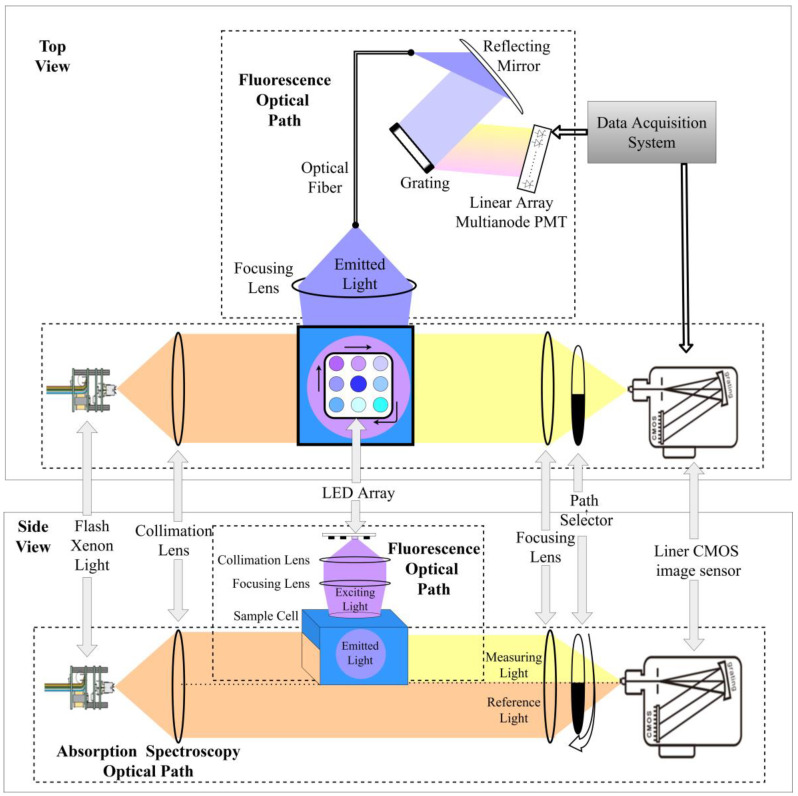
Schematic diagram of the integrated absorption–fluorescence spectroscopy system optical design.

The design schematic of the absorption–fluorescence spectroscopic measurement system is shown in Figure 1. The system consists of two parts: the absorption spectroscopic measurement path and the three-dimensional fluorescence spectroscopic measurement path. The absorption and fluorescence paths are arranged in a cross configuration to avoid mutual interference between the two paths. The absorption detection path uses a xenon flash lamp as the light source. After passing through a collimating lens, part of the light passes through the sample cell, and the other part passes through an empty cavity below the sample cell. The light passing through the sample cell is called the measurement light and the light passing through the cavity below the sample cell is called the reference light. The ratio of the two intensities at different wavelengths is the absorbance spectrum value of the solution. A focusing lens and a rotatable optical path selector are used at the receiving end to switch between the upper and lower light paths. When the shutter of the receiver’s light path selector is turned down, the linear array CMOS detector receives the light spot formed by the measuring light, while when the shutter is turned up, the detector receives the light spot formed by the reference light. The absorption spectroscopic measurement system can directly obtain the full absorbance spectrum information of the solution in the wavelength range of 200–700 nm in one test.

The three-dimensional fluorescence spectroscopic measurement path is mainly composed of an LED array light source, a collimating and focusing lens group, a grating, and a 32-channel array photomultiplier tube. The three-dimensional fluorescence signal requires an excitation light source composed of light sources of different wavelengths. Therefore, an LED light source array composed of single-color and well-behaved LED beads with wavelengths from 255 nm to 380 nm was used to form the LED light source array. The light emitted by the LED light source is collimated and focused before being irradiated onto the quartz sample cell. This light path is called the excitation light path. The emitted fluorescence produced by the solution after excitation is coupled into the optical fiber through the focusing lens group and then irradiated onto the grating after collimation. After dispersion, the light is accurately irradiated onto the 32-channel photomultiplier tube array according to the wavelength, and the photomultiplier tube can convert the emitted light signal of each wavelength into a current signal. By quickly scanning and collecting the current signals on each photomultiplier tube and using a transimpedance amplifier and high-speed signal acquisition circuit, full-spectrum fluorescence detection from 200–700 nm can be achieved.

This system provides a simultaneous acquisition of spectral information, which can improve the efficiency of spectral measurement. The merged optical path structure allows the measurement light sources for absorption spectroscopy and fluorescence spectroscopy to act on the same sample cell during the measurement process, thus saving the sample replacement process. This instrument completes the integration of spectral information acquisition by simultaneously obtaining the absorbance and fluorescence spectra of the sample in one measurement.

### 2.2. D Gabor Transform

Traditional methods for extracting features from three-dimensional fluorescence spectra mainly use linear transformation algorithms such as principal component analysis (PCA) and independent component analysis (ICA). Although these algorithms can directly extract spectral features, they often use dimensionality reduction, which results in information loss and poor interpretability of the feature matrix, affecting the effectiveness of feature fusion.

To address this issue, 2D Gabor transform can be used for feature extraction of three-dimensional fluorescence spectra [33]. As an extension of Fourier transform, 2D Gabor transform can be used to extract texture features, edge features, contour features, and other information from images [34]. The kernel function of 2D Gabor transform is a sine and cosine waveform with different frequencies and phases. By combining these kernel functions with different frequencies and phases, 2D Gabor transform can represent an image as a linear combination of responses from multiple kernel functions with different frequencies and phases. By performing 2D Gabor transform on an image, contour and texture information can be extracted, and a more complete representation of the image features can be obtained. In image recognition and classification tasks, this feature information can serve as the basis for subsequent analysis and decision making, thus improving the efficiency and accuracy of image analysis [35].

A 2D Gabor transform kernel is represented as a product of a Gaussian kernel and a sinusoidal function in the spatial domain [36], where the Gaussian kernel is primarily used to represent the temporal locality of the kernel function. Specifically, the shape of the Gaussian kernel determines the radius of the 2D Gabor transform kernel, i.e., the space length of the processed signal. At the same time, the variance of the Gaussian kernel determines the strength of temporal locality, i.e., the sensitivity of the kernel function to the signal; the sine function is used to define the frequency characteristics of the kernel function and to provide frequency response to the input signal. This 2D Gabor transform can be represented in complex form as:

(1) $$g\;(x,\;y;\;\lambda ,\;\theta ,\;\psi ,\;\sigma ,\;\gamma )=e^{\frac{-{x^{\prime }}^{2}+\gamma^{2}y{’}^{2}}{2\sigma^{2}}}\;\cdot \;e^{i\left(2\pi \frac{x^{\prime }}{\lambda }+\psi \right)}$$

and decompose the kernel function into real and imaginary parts:

(2) $$\left\{\begin{array}{c} g_{real}\;(x,\;y;\;\lambda ,\;\theta ,\;\psi ,\;\sigma ,\;\gamma )=e^{\frac{-{x^{\prime }}^{2}+\gamma^{2}y{’}^{2}}{2\sigma^{2}}}\cos\left(2\pi \frac{x^{\prime }}{\lambda }+\psi \right)\\ g_{imag}\;(x,\;y;\;\lambda ,\;\theta ,\;\psi ,\;\sigma ,\;\gamma )=e^{\frac{-{x^{\prime }}^{2}+\gamma^{2}y{’}^{2}}{2\sigma^{2}}}\sin\left(2\pi \frac{x^{\prime }}{\lambda }+\psi \right) \end{array}\right.$$

where

(3) $$\left\{\begin{array}{c} x^{\prime }=x\cos\theta +\mathrm{ysin}\theta \\ y^{\prime }=-x\sin\theta +\mathrm{ycos}\theta \end{array}\right.$$

where λ is the wavelength of the sinusoidal function, which directly affects the response scale of the 2D Gabor transform. θ is the response direction of the 2D Gabor transform kernel function, and features in the image in this direction will be extracted. Therefore, θ ranges from 0 to 2π. σ represents the bandwidth, which is the variance of the Gaussian filter, and is taken as 2π. γ is the aspect ratio of the kernel function and determines the eccentricity of the kernel function shape. When γ = 1, the 2D Gabor transform kernel function is circular. When γ = 0, the filter function is a straight line. When γ is between 0 and 1, the envelope of the filter kernel function is an ellipse, and the ellipse shape elongates with the internal parallel stripe direction. Typically, this value is set to 0.5. ψ is the phase parameter of the tuning function and ranges from −π to π. For example, when ψ is 0 or π, the filter kernel function is symmetric about the vertical axis. When ψ is −π/2 or π/2, it is symmetric about the origin.

The size of the 2D Gabor transform kernel in the spatial domain is determined by θ, σ, and γ. Since the 2D Gabor transform contains a Gaussian function, and in the spatial domain, it is an ellipse (or circle), according to the 3σ law, the short-axis radius of the kernel function in polar coordinates will not exceed 3σ. The shape of the kernel function ellipse, i.e., the scaling factor relative to the circle, can be controlled by the eccentricity γ. Therefore, 3σ/r represents the long axis of the outer envelope ellipse of the 2D Gabor transform. As a result, the maximum lengths of the x-axis and y-axis projections of the 2D Gabor transform kernel in the spatial domain are obtained as follows:

(4) $$\left\{\begin{array}{c} X_{\max}=\max\left(\left|3\right.\left.\sigma \;\cdot \;\cos\theta \right|,\;\left|3\frac{\sigma }{\lambda }\;\right.\left.\cdot \;\sin\theta \right|\right)\\ Y_{\max}=\max\left(\left|3\right.\left.\sigma \;\cdot \;\sin\theta \right|,\;\left|3\frac{\sigma }{\gamma }\;\right.\left.\cdot \;\cos\theta \right|\right) \end{array}\right.$$

therefore, the square-shaped spatial domain size of the 2D Gabor transform kernel is:

(5) $$K=\max\left(Y_{\max},\;X_{\max}\right)$$

different kernel orientations θ affect the feature direction extracted via 2D Gabor transform. When θ is aligned with the texture direction in the image, it can maximize the response of 2D Gabor transform and highlight the texture features in spectral images. On the other hand, when θ is not aligned with the texture direction, the response of 2D Gabor transform to three-dimensional fluorescence spectra is reduced, making it difficult to extract spectral features. Therefore, to maximize the extraction of texture features, the θ of 2D Gabor transform needs to be selected according to the texture direction in the image, and the selected kernel orientation is symmetric about π/2. The corresponding feature extraction results are also symmetric about π/2.

### 2.3. Absorption–fluorescence Spectral Feature Fusion Algorithm

**Figure 2:**
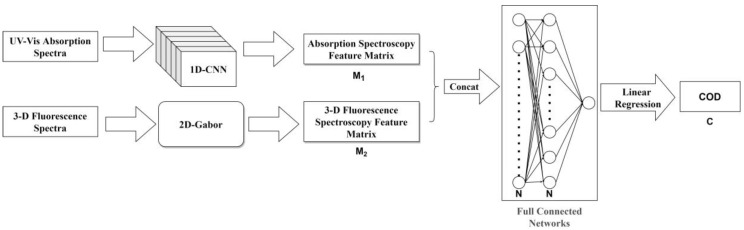
Modeling and Retrieval Process of Water COD Based on Absorption–Fluorescence Spectral Feature Fusion.

The focus of feature fusion research is on how to extract UV-Vis absorption spectrum and three-dimensional fluorescence spectrum features, establish a fused feature matrix, separately extract features from the absorption spectrum and the three-dimensional fluorescence spectrum to produce two feature matrices, combine the data from the two matrices, model and analyze the fused results, and ultimately obtain the COD retrieval result of the fused spectrum. The process of spectral feature fusion is shown in Figure 2 and includes the following steps:

(1) Absorption spectrum feature extraction: The absorption spectrum is used as input data, and its feature matrix is obtained through 1-D CNN. The input data for the absorption spectrum are represented as:

(6) $$X_{\mathrm{abs}}\;\in {\;R}^{n\times 1},\;$$

where $R^{n\times 1}$ represents a UV-Vis absorption spectrum data of length n. 

The 1-D CNN feature extraction process can be represented as

(7) $$M_{1}{=f}_{1}\left(X_{\mathrm{abs}}\right),\;M_{1}\;\in {\;R}^{m_{1}{\times c}_{1}},\;$$

where $M_{1}$ represents the extracted absorption spectrum feature matrix, $f_{1}$ represents the 1-D CNN algorithm for absorption spectral feature extraction, $m_{1}$ represents the number of rows in the feature matrix, and $c_{1}$ represents the number of columns in the feature matrix.

(2) Three-dimensional fluorescence spectrum feature extraction: The three-dimensional fluorescence spectrum is used as input data, and a feature matrix is obtained through an image feature extraction algorithm. The process of three-dimensional fluorescence spectrum feature extraction can be represented as:

(8) $$M_{2\;}{=f}_{2}\left(X_{\mathrm{fluor}}\right){,\;M}_{2}\in R^{\left\{m_{2}{\times c}_{2}\right\}}{,\;X}_{\mathrm{fluor}}\in R^{\left\{n\;\times m\;\times k\right\}},\;$$

where $M_{2}$ is the extracted three-dimensional fluorescence spectrum feature matrix, $X_{\mathrm{fluor}}$ is the input three-dimensional fluorescence spectrum data, $f_{2}$ represents the three-dimensional fluorescence spectrum feature extraction algorithm, $m_{2}$ represents the number of rows in the feature matrix, $c_{2}$ represents the number of columns in the feature matrix, and n, m, and k represent the emission wavelength, excitation wavelength, and fluorescence intensity of the three-dimensional fluorescence spectrum, respectively.

(3) Feature concatenation: The absorption spectrum feature matrix and the three-dimensional fluorescence spectrum feature matrix are concatenated to obtain a new feature matrix. The concatenation process is represented as:

(9) $$M_{3}=\left[M_{1}{,M}_{2}\right],\;M_{3}\in R^{m_{3}\times \left(c_{1}{+c}_{2}\right)},\;$$

where $\left[\cdot ,\cdot \right]$ represents the concatenation operation. To complete the concatenation operation, the number of row vectors or column vectors in the extracted feature matrix must be consistent: $m_{3}={\;m}_{1}={\;m}_{2}$.

(4) Feature fusion: The concatenated feature matrix is used as input, and a feature value is obtained through feature extraction and dimensionality reduction via a fully connected layer. This process can be represented as

(10) $${Z=f}_{3}\left(M_{3}\right),\;Z\in R^{N\times 1},\;$$

where $f_{3}$ represents the fully connected layer and N represents the dimension of the hidden layer in the fully connected layer.

(5) Regression analysis: The feature value is linearly fitted with the COD label value C to establish a COD retrieval model, which can be represented as

(11) $${C=f}_{4}\left(Z\right),\;$$

where $f_{4}$ represents the linear regression model. 

In the context of feature extraction and fusion, absorption spectra and three-dimensional fluorescence spectra are two types of spectral data with different dimensions. However, unbalanced feature matrix dimensions create problems when performing feature extraction and fusion. Typically, absorption spectra are represented by a one-dimensional vector, whereas three-dimensional fluorescence spectra are represented by a two-dimensional matrix. In order to preserve the important spectral features while reducing the impact of unbalanced dimensions, an appropriate dimensionality reduction algorithm is applied to the feature matrix of three-dimensional fluorescence spectra to decrease its dimensionality difference with the feature matrix of absorption spectra. Additionally, there is a need for further research on strategies and algorithms for feature fusion which combines absorption spectra and three-dimensional fluorescence spectra feature matrices to improve the accuracy of COD concentration retrieval.

**Figure 3:**
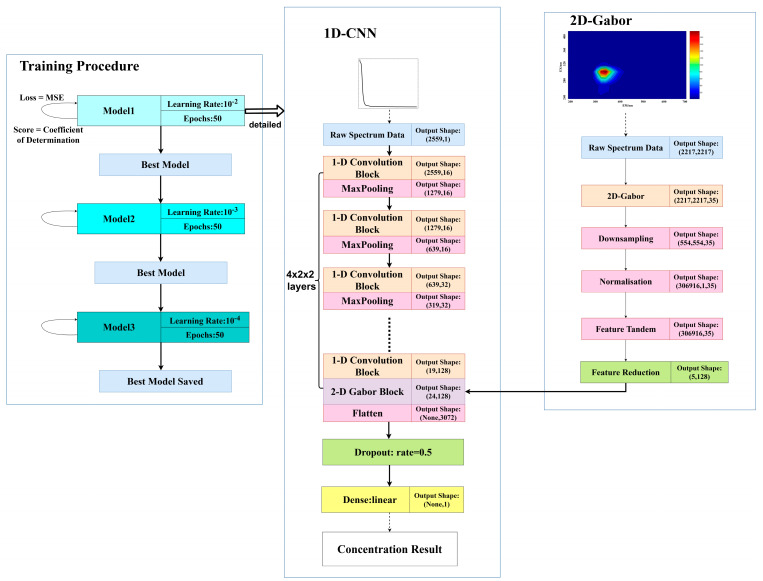
Fusion CNN structure designed for COD retrieval based on absorbance-fluorescence spectra.

Inspired by the FractalNet structure [37], a fusion convolutional neural network (Fusion CNN) is developed for absorbance–fluorescence spectral analysis. The network structure is shown in Figure 3. The neural network still consists of 8 layers of convolutional blocks, each of which is followed by a max-pooling layer. After passing through the 8th layer of the convolutional block, the absorbance spectrum produces a 19 × 128 absorbance spectral feature matrix. Then, the fluorescence spectrum is inputted and a 2D Gabor transform is used to extract the 5 × 128 fluorescence spectral feature matrix. The fluorescence and absorbance spectral feature matrices are concatenated to obtain a 24 × 128 fusion spectral feature matrix. The matrix is then flattened and subjected to random dropout before being passed through a fully connected layer with a linear activation function to fuse the fluorescence and absorbance spectral data and obtain the final COD retrieval result.

The input of this network consists of absorption spectrum data with a length of 2559 × 1 and a fluorescence spectrum data with a size of 2217 × 2217. Eight convolutional layers are utilized to extract features from the absorption spectrum, resulting in a 19 × 128 absorption spectrum feature matrix, which is then subject to dimensionality reduction via 2D Gabor transform, equidistant feature mapping, and downsampling, ultimately yielding a 5 × 128 fluorescence spectrum feature matrix. The fluorescence spectrum feature matrix is concatenated with the absorption spectrum feature matrix to obtain a fusion spectrum feature matrix of size 24 × 128. The fusion spectrum feature matrix is flattened into a one-dimensional vector using the Flatten layer, allowing each feature value to participate in the processing of the fully connected layer. Before the data enter the fully connected layer, a Dropout layer is employed to randomly discard 50% of the convolutional network nodes to prevent model overfitting. The output layer is a fully connected layer with a single neuron that uses a linear activation function, which is used to predict the COD corresponding to the input spectrum. In this network, the fully connected layer can make full use of the fusion spectrum feature information extracted from the convolutional layers and 2D Gabor transform to perform spectral data fusion via parameter learning, thereby improving the accuracy and robustness of spectral data processing.

## 3. Results

### 3.1. Pre-Processing

In this paper, three common amino acid organic compounds in water were selected as the research objects, namely phenylalanine, tyrosine, and tryptophan. Different concentrations of solutions were prepared by dissolving the pure solids of these organic compounds into deionized water. From a structural perspective, these organic compounds can be mainly divided into two categories: single-ring and two-ring structures and bicyclic structures. The chemical method for COD measurement employed the Lovibond RD125 COD digester and the Lovibond Multidirect multi-parameter photometer. The instrument’s algorithm for COD measurement is based on the method of potassium dichromate and is widely used in the field of water quality analysis.

Assuming that all organic matter is oxidized to CO_2_ and H_2_O, the oxidation process of organic matter can be simplified as:

$$C_{m}H_{n}O_{l}+\left(2m+\frac{n}{2}-l\right)O=mCO_{2}+\frac{n}{2}H_{2}O$$

Therefore, the conversion formula of oxidant mass consumed per unit mass of organic matter can be obtained as:

$$\alpha_{COD}=\left(2m+\frac{n}{2}-l\right)\cdot Mr\left(O\right)/Mr\left(C_{m}H_{n}O_{l}\right)$$

where $\alpha_{COD}$ is the conversion coefficient of COD, m is the number of carbon atoms in organic matter, n is the number of hydrogen atoms in organic matter, and l is the number of oxygen atoms in organic matter. $Mr\left(O\right)$ represents the molecular weight of oxygen atoms and $Mr\left(C_{m}H_{n}O_{l}\right)$ represents the molecular weight of organic matter. The theoretical conversion coefficients of COD for four kinds of organic matter are calculated using this formula, and the actual conversion coefficients of COD measured using the potassium dichromate method for each kind of organic matter are obtained through measurements using the Lovibond RD1COD digester and Lovibond Multidirect multi-parameter colorimeter. The results are shown in Table 1. 

The COD test results of the solution with different components were used as the true values (label values) of the dataset. Then, the absorption spectra dataset of phenylalanine, tryptophan, and tyrosine mixed at different concentrations were measured using the designed hardware system. The dataset structure is shown in Table 2. The labeled data used for the mixed solution absorption spectra dataset were calculated based on the actual COD conversion coefficients measured in Table 1. The COD values of the solution were calculated based on the concentration and COD conversion coefficients of different amino acid substances in the mixture, and the COD label values were sorted in ascending order.

**Figure 4:**
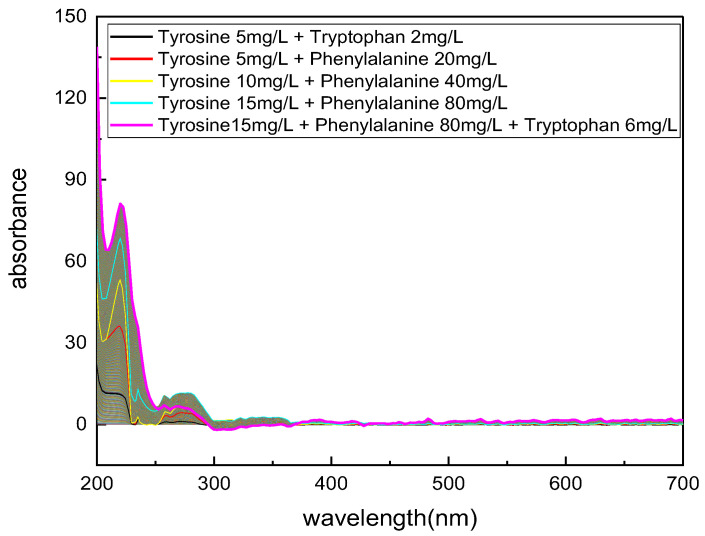
The absorption spectroscopy dataset with COD label ranging from 0 to 146.57 mg/L after data augmentation.

After the annotation of the COD labels in the dataset, further data augmentation was performed on the blank group absorbance spectra of COD values. The spectral interpolation algorithm was used to fill the missing absorbance spectra data points, with a data interval of 0.01 mg/L COD label values. For example, for a mixed solution of 5 mg/L tyrosine and 2 mg/L tryptophan, the corresponding COD label is 9.34 mg/L, while for a mixed solution of 10 mg/L tyrosine and 4mg/L tryptophan, the corresponding COD label is 18.78 mg/L. Therefore, the number of absorbance spectra groups to be filled is (18.78–9.34)/0.01 = 944. The spectral interpolation algorithm used for the absorbance spectra is the same as that used in Section 3.2, which uses the cubic spline interpolation algorithm in the “interpolate.interp1d()” function of the “SciPy” library in Python. The interpolation effect of the mixed amino acid solution absorbance spectra is shown in Figure 4.

**Figure 5:**
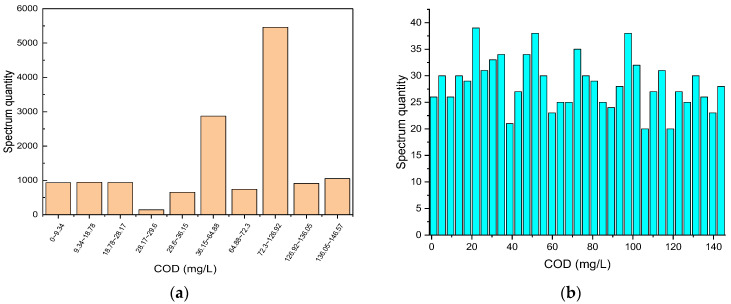
The distribution of the absorption spectra dataset for amino acid mixtures (**a**) and distribution of 1000 test data (**b**).

The final dataset contains 14,657 absorbance spectra data after data augmentation, with COD label values ranging from 0 to 146.57 mg/L, and their distribution is shown in Figure 5a. The test set was randomly sampled from the total dataset using the “sample” function in the “pandas” library in Python, with a sample size of 1000. The data distribution of the test set is shown in Figure 5b.

**Figure 6:**
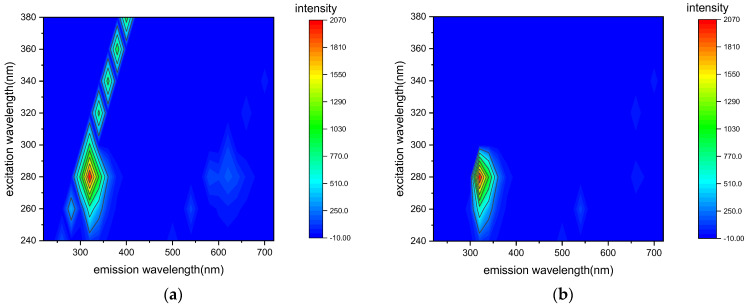
Three-dimensional fluorescence spectra of a 15 mg/L tyrosine solution before (**a**) and after (**b**) scatter correction.

To input fluorescence spectral data into a model, it is necessary to remove scattering interference from the measured fluorescence spectral data, as shown in Figure 6a. The Delaunay triangulation interpolation method is used to remove the scattering interference by measuring the three-dimensional fluorescence spectrum of deionized water, determining the specific position and width of the scattering band. The scattering-corrected three-dimensional fluorescence spectroscopy of a 15 mg/L tyrosine solution is shown in Figure 6b, where the scattering information is effectively removed, and the feature peak information is preserved.

**Figure 7:**
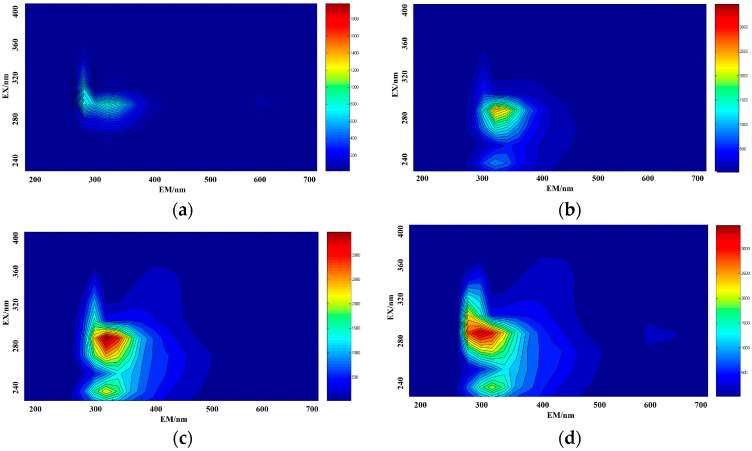
The three-dimensional fluorescence spectra of the mixed solutions of tyrosine–tryptophan (**a**), tyrosine–phenylalanine (**b**), tryptophan–phenylalanine (**c**) and tryptophan–tyrosine–phenylalanine (**d**).

Mixed amino acid solutions were prepared according to the proportions given in Table 1. Three-dimensional fluorescence spectra were measured for each sample. Some of the measured results are shown in Figure 7, where Figure 7a represents the fluorescence spectrum data of a mixed solution containing 5 mg/L tyrosine and 2 mg/L tryptophan, Figure 7b represents the fluorescence spectrum data of a mixed solution containing 5 mg/L tyrosine and 20 mg/L phenylalanine, Figure 7c represents the fluorescence spectrum data of a mixed solution containing 2 mg/L tryptophan and 40 mg/L phenylalanine, and Figure 7d represents fluorescence spectrum data of a mixed solution of 15 mg/L Tyrosine, 80 mg/L phenylalanine, and 6 mg/L tryptophan.

**Figure 8:**
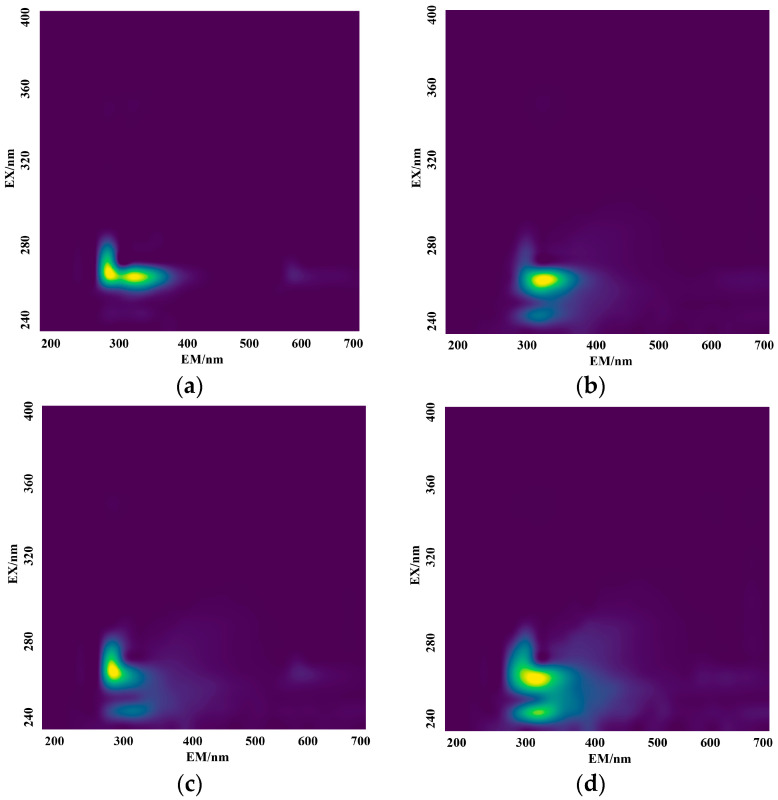
Interpolated 3D fluorescence spectrum image of mixed solution of tyrosine–tryptophan (**a**), tyrosine–phenylalanine (**b**), tryptophan–phenylalanine (**c**) and tryptophan–tyrosine–phenylalanine (**d**).

In order to interpolate the measured three-dimensional fluorescence spectroscopy data, the cubic spline non-linear interpolation method was employed, resulting in an expansion of the original 32 × 9-pixel three-dimensional fluorescence image to 2217 × 2217. The interpolated three-dimensional fluorescence spectroscopy data of the different amino mixed solution are shown in Figure 8.

### 3.2. Fluorescence Feature Extraction

**Figure 9:**
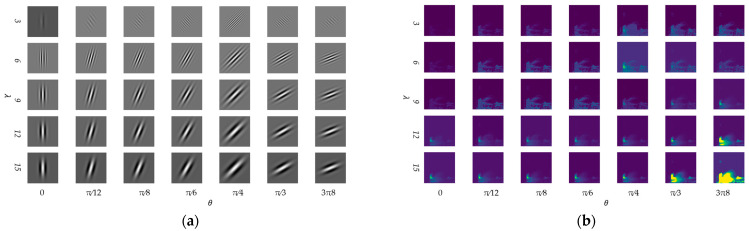
When λ = 3, 6, 9, 12, 15, θ = 0, π/12, π/8, π/6, π/4, π/3, 3π8, 2D Gabor kernel function (**a**) and corresponding spectral responses (**b**).

Through experiments, it was found that when the orientation of the kernel function is π/2, the kernel function cannot extract effective texture features from the fluorescence spectrum. Therefore, the parameter range of the kernel function orientation angle θ was further determined to be [0, π/2). Different wavelengths λ affect the scale of feature extraction, with smaller wavelengths capturing high-frequency texture details such as edges, and larger wavelengths capturing lower-frequency image information such as contour structures. In order to comprehensively extract fluorescence spectrum features at different scales and wavelengths, the kernel function wavelength was set to λ = 3, 6, 9, 12, 15, and the kernel function orientation θ was set to 0, π/12, π/8, π/6, π/4, π/3, and 3π8, resulting in the kernel functions shown in Figure 9a and their corresponding spectral responses shown in Figure 9b. As shown in the figure, under the above scales and kernel function angles, the 2D Gabor transform obtained effective spectral responses.

Next, the concatenation and flattening of the features extracted from the 35 2D Gabor operators are introduced. Since 2D Gabor filters are usually a group of filters and the filtering results are a combination of multiple feature matrices, to simplify the expression of feature matrices, the filtering results of each direction or scale of the filter kernel can be flattened in the direction of the excitation spectrum to obtain a one-dimensional matrix. Then, all the one-dimensional matrices of the 2D Gabor filter results are concatenated and flattened to obtain the final feature matrix. The fluorescence spectra of amino acid mixed organic solutions of different concentrations and types were used for this study, and the same parameters were applied to the 2D Gabor filter. Each filtering result is a grayscale image with a gray value range of 0–255 for each pixel. 

**Figure 10:**
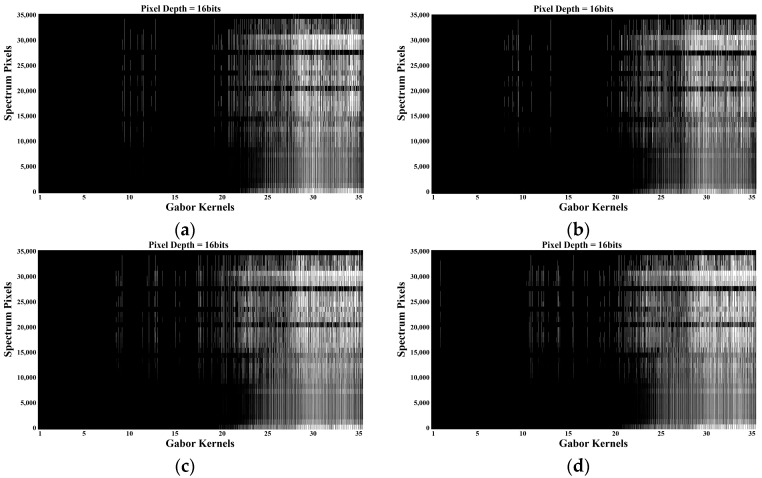
Fluorescence spectral feature matrix of mixed amino acid solution of tyrosine–tryptophan (**a**), phenylalanine–tyrosine (**b**), phenylalanine–tryptophan (**c**) and phenylalanine–tyrosine–tryptophan (**d**).

The fluorescence spectral feature matrix is shown in Figure 10. The x-axis in Figure 10 represents the different Gabor kernel functions selected from Figure 9, totaling 35 kernels. The y-axis represents the pixel positions of the feature images extracted by each Gabor kernel function on the fluorescence spectrum (each image from Figure 9b). The color blocks (z-axis) within represent the spectral responses at the corresponding pixel positions, where pure black indicates no response (z = 0) and pure white indicates the maximum response (z = 255).

Based on the previous chapters, we know that to successfully concatenate the fluorescence and absorption spectral feature matrices, it is necessary to perform a dimensionality reduction on the concatenated 2D Gabor transformed feature matrix in order to match the absorption spectral feature matrix and prevent imbalanced feature data between fluorescence and absorption spectra. Various dimensionality reduction algorithms have been proposed in current research, and in this study, the mainstream dimensionality reduction algorithms are applied to the concatenated fluorescence feature matrix. The coefficient of variation ($C_{v}$) and the mean of $C_{v}$s in each feature direction of the reduced feature matrix are calculated to evaluate the degree of dispersion of the extracted feature matrix on each feature vector, and the algorithm’s running time is also considered to screen the dimensionality reduction algorithm. The calculation of the average coefficient of variation ($\bar{C_{v}}$) is shown as follows:

(12) Cv¯= ∑i=1nσiμin,

where *n* = 35 is the total number of feature vectors, $\sigma_{i}$ is the variance of each feature vector in the feature matrix, and $\mu_{i}$ is the mean of each feature vector. The larger the value of the average coefficient of variation, the greater the difference between the extracted feature vectors, indicating that the extracted feature vectors are more representative. The evaluated feature dimensionality reduction algorithms mainly include Principal Component Analysis (PCA), Fast Independent Component Analysis (FastICA), Isometric Feature Mapping (Isomap), Locally Linear Embedding (LLE), Gaussian Random Projection (GRP), and Sparse Random Projection (SRP). The number of principal components extracted by each algorithm is set to 20. The result is shown in Table 3.

In summary, Isomap is the best-performing dimensionality reduction algorithm, with an average coefficient of variation of 1.402 for each feature vector, representing good distinguishability between each feature vector. Its program runtime is 0.802 s, which satisfies the real-time requirements of the fluorescence spectral feature extraction process.

**Figure 11:**
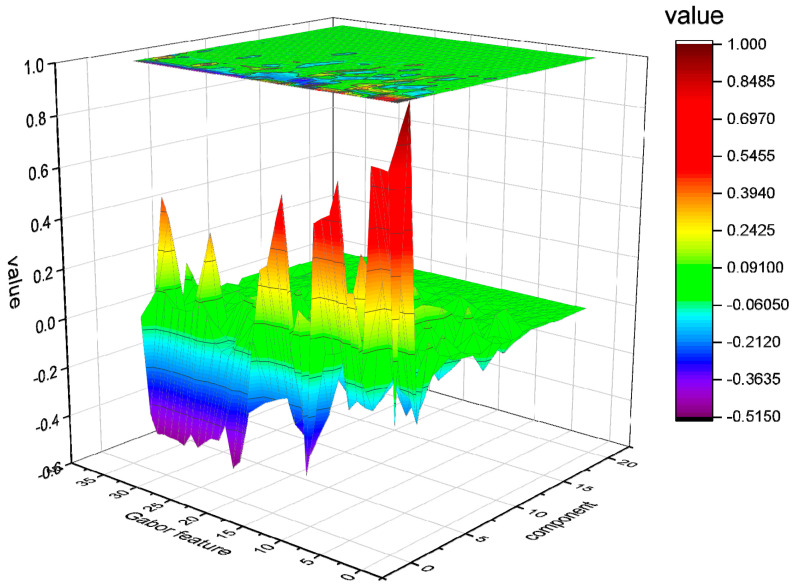
The tyrosine–tryptophan fluorescence spectral feature matrix obtained after dimensionality reduction.

The tyrosine–tryptophan fluorescence spectral feature matrix obtained after dimensionality reduction is shown in Figure 11. The size of the feature matrix is reduced from 306,916 × 35 to 20 × 35, where 35 represents the number of 2D Gabor filter kernels, i.e., the total number of feature vectors, and 20 represents the 20 principal components obtained after dimensionality reduction for each feature vector. From the figure, it can be seen that most of the information in the reduced feature matrix is concentrated between the first and tenth principal components. Since the convolutional neural network extracts a feature matrix of size 19 × 128 after processing through a convolutional layer, it is necessary to transform the shape of the fluorescence spectral feature matrix after dimensionality reduction into n × 128. To achieve this, further feature selection needs to be performed on the 20 × 35 feature matrix to obtain a new feature matrix consisting of 640 elements. Next, the processed matrix data are flattened and reconstructed into a matrix shape, resulting in fluorescence spectral data 5 × 128 in size. These data can be directly concatenated with the feature matrix of the absorption spectrum and input into the next fully connected layer of the convolutional neural network for data fusion training.

### 3.3. Network Training

To train the model more efficiently, a small server with two Geforce RTX 3090 graphics cards (Nvidia Corporation, Santa Clara, CA, USA), an Intel^®^ Xeon^®^ Silver 4210 CPU with a clock frequency of 2.20GHz (Intel Corporation, Santa Clara, CA, USA), and 32GB RAM was employed to train the model. Tensorflow 2.6.0 and CUDA 11.2 frameworks were utilized to fully exploit the performance advantages of the hardware and improve the training efficiency and accuracy. The model was trained for three cycles, with each cycle consisting of 50 epochs. The Adam optimization algorithm was used to optimize the network parameters, with its learning rate exponentially decreasing during the training process to accelerate network convergence. At the end of each stage, the model saved the best training results for subsequent testing and retrieval. The mean square error (MSE) function was still used as the loss function during training, which measured the error between the network predicted output and COD label values.

**Figure 12:**
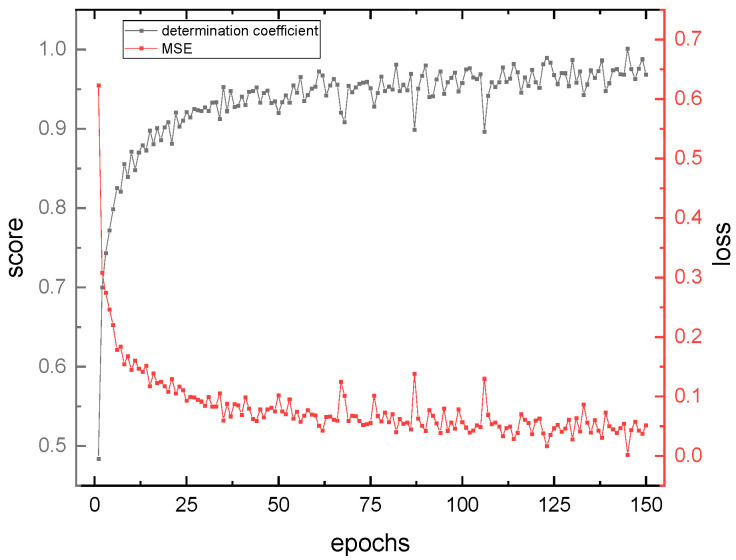
The variation in the model score and loss value during the training cycles of Fusion CNN.

During the training process of the Fusion CNN network, the coefficient of determination (R-Square) was used as the model score and the mean squared error (MSE) was used as the model loss. The changes of these two parameters during the training process are shown in Figure 12.

The neural network model for COD retrieval based on absorption–fluorescence spectral feature fusion was evaluated using R-Square and MSE as evaluation indices. R-Square reflects the linear correlation between the true and inverted COD values during the model training process, and the Score curve showed a rapid rise from 0, fluctuated significantly between 50 and 100 epochs, and stabilized around 1 after 150 epochs. MSE was used to evaluate the effectiveness of the neural network training from the perspective of Loss, and the Loss value decreased rapidly within the first 25 epochs and changed relatively flatly, with significant fluctuations between 50 and 100 epochs, stabilizing around 0 after 100 epochs. The results show that the neural network model exhibits high accuracy and small errors in the retrieval of COD for fusion spectra, and the final trained model is stable and reliable. Overall, the absorption–fluorescence feature fusion spectrum neural network model quickly converges during the training process.

Table 4 presents the architecture parameters of the final built absorption–fluorescence feature fusion spectral neural network model (Fusion CNN). The network training parameters consist of 733,729, with a total of 73 layers. The input data are the UV-visible absorption spectrum data ranging from 200–700 nm and corresponding 3D fluorescence spectrum data with an excitation wavelength of 250–400 nm and an emission wavelength of 200–700 nm. The absorption spectrum data used the oversampled data from the CMOS image sensor, while the fluorescence spectrum data used the enhanced interpolation data. Therefore, the absorption spectrum input dimension is 2559 × 1, and the fluorescence spectrum input dimension is 2217 × 2217.

The advantage of the fractal-structured fusion CNN method proposed in this article lies in its incorporation of the feature information from three-dimensional fluorescence spectra to supervise the network training of absorption spectra, thereby improving the accuracy of the network in the retrieval of mixed organic compound solution COD. The strength of this neural network lies in its dual-channel parallel convolutional structure, which accelerates the training speed of the network. For a comparison of the network’s training speed, please refer to our previously published article [38]. Another advantage is the lightweight nature of the network, as its training parameters are relatively small compared to those of other networks. The parameter comparison of the network’s scale is shown in Table 5.

### 3.4. COD Retrieval

**Figure 13:**
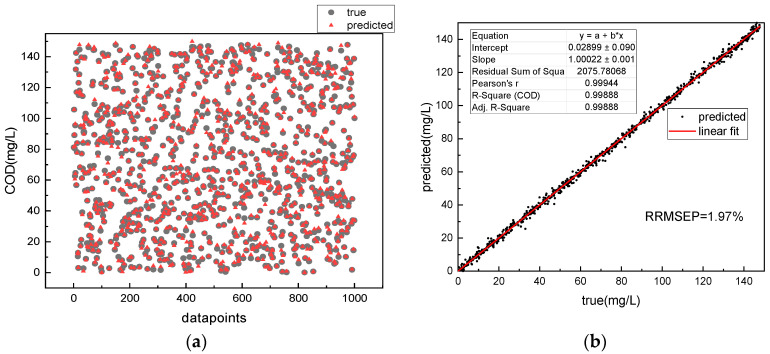
Retrieval of COD in amino acid mixed solution based only on 1-D CNN and absorption spectra: (**a**) retrieval results of COD and (**b**) retrieval precision evaluation.

**Figure 14:**
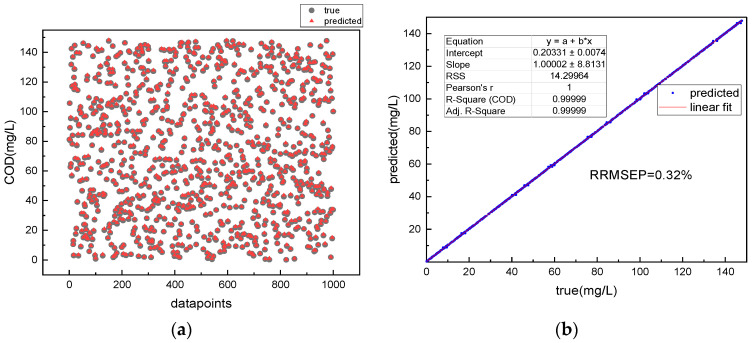
Retrieval of COD in amino acid mixed solution based on Fusion CNN and absorption–fluorescence Spectra: (**a**) retrieval results of COD and (**b**) retrieval precision evaluation.

After the training process is completed, the model with the highest R-Square is saved and used as the final model. In order to further evaluate the performance of the model, the absorption-three-dimensional fluorescence spectra are input into the trained model to retrieve COD values, which are then compared with the true COD values. Figure 13a shows the COD retrieval results of the 1D CNN algorithm for a single absorption spectrum of amino acid mixtures. It can be seen from the figure that many triangles do not overlap with the corresponding circles, indicating a large deviation between the retrieved and true values. Figure 14a shows the retrieved COD values and their corresponding true values. The circles represent the true values and the triangles represent the retrieved values. The more the triangles and circles overlap, the closer the retrieved values are to the true values. Most of the triangles in the figure overlap with the circles By comparing Figure 13a and Figure 14a, it is found that the absorption–fluorescence spectra combined with the Fusion CNN algorithm improve the accuracy of COD retrieval for amino acid mixtures.

To further quantify the retrieval accuracy of the model, linear analysis is performed on the retrieved COD values and the true values. The higher the COD retrieval accuracy, the better the linearity between the true and retrieved values, and the closer the slope of the fitted line is to 1. The retrieval accuracy can be quantitatively evaluated by R-Square and the slope of the fitted line. Figure 13b shows the linear fitting result of the retrieved and true values using the 1D CNN model for a single absorption spectrum of amino acid mixtures. Figure 14b shows the linear fitting result of the retrieved and true values using the Fusion CNN model. Compared with Figure 13b, it can be seen that the retrieval result of the 1D-CNN algorithm for amino acid mixtures with a single absorption spectrum is linearly related to the true values, but the data points are scattered on the fitted line, and some data points deviate from the fitted line. However, the scatter plot of the Fusion neural network model shows good linearity, and all points are distributed near the line with a slope of 1. The fitting slope of the Fusion CNN between the retrieval value and the true value was 1.00002, with an R-Square of 0.9999, and the residual sum of squares (RSS) between the retrieval value and the true value was only 14 (mg^2^∙L^−2^). In contrast, the fitting slope of the 1D-CNN model based on single absorption spectra between the retrieval value and the true value was 1.00022, with an R-Square of 0.9988, and the RSS between the retrieval value and the true value was 2076 (mg^2^∙L^−2^). 

**Figure 15:**
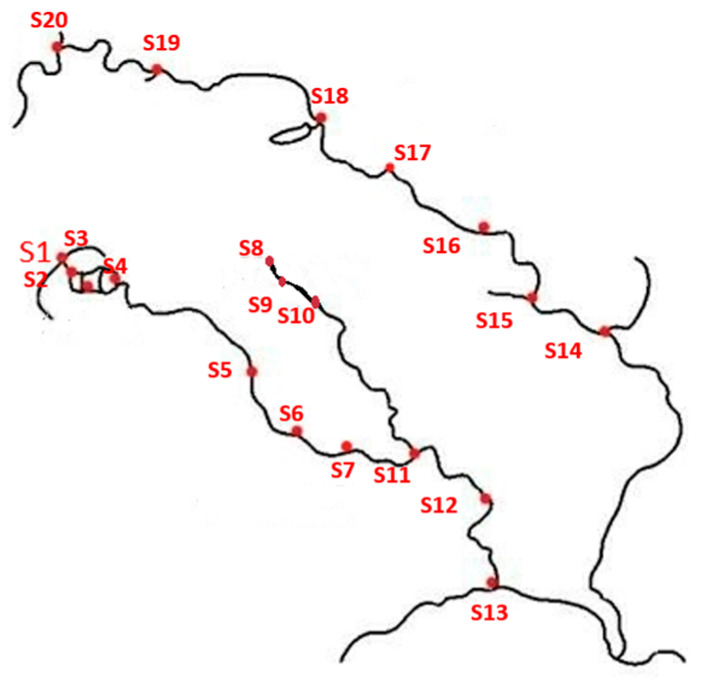
Distribution of sample points.

In addition, the relative root mean square error of the model’s prediction (RRMSEP) was calculated. The results showed that the RRMSEP of the 1D-CNN for the retrieval of COD for mixed amino acid solution was 1.97%, while that of the Fusion CNN for COD retrieval was only 0.32%, a decrease of 84%. The acceptable range of relative error between the COD retrieval value and the true value was defined as 5%. The comparison of COD retrieval accuracy between 1D-CNN and Fusion CNN for the test set is shown in Figure 15. The accuracy of 1D-CNN was 85%, while that of Fusion CNN for the retrieval of COD in mixed amino acid solution based on absorption spectra was 98%, which was 13% higher than that of 1D-CNN. These results indicate that compared with the 1D-CNN based on single absorption spectra, the Fusion CNN algorithm based on absorption-three-dimensional fluorescence spectra has higher accuracy in COD retrieval for mixed amino acid solution.

The accuracy of the algorithm for the COD retrieval of complex organic matter solutions was verified using the measured absorption–3D fluorescence spectrum data as the model input. The results showed that the COD retrieval value-real value residual sum of squares (RSS) of the absorption-3D fluorescence spectrum combined with the Fusion CNN algorithm was only 14 (mg^2^·L^−2^), and the relative root mean square error (RRMSEP) was 0.32%, with a COD retrieval accuracy of 98%. In contrast, the COD retrieval value-real value RSS using single absorption spectrum combined with the 1D-CNN algorithm was as high as 2075 (mg^2^·L^−2^), with an RRMSEP of 1.97%, and a COD retrieval accuracy of only 85%. Thus, the Fusion CNN algorithm based on the absorption-3D fluorescence spectrum has higher accuracy in COD retrieval.

When applying this method to actual water bodies, it is necessary to collect spectral samples from real water bodies and further train the network based on laboratory data. The proposed supervised learning network can utilize the fluorescence spectral feature extraction algorithm to extract information about the organic compound composition. This composition information can then supervise the training process of the absorption spectral neural network, thereby addressing the issues of spectral peak overlap and low learning accuracy in single absorption spectral methods for predicting COD. In practical applications, in order to obtain more accurate learning results, it is important to increase the training dataset with spectral data from different scenarios of actual water samples, thus enhancing the model’s generalization ability.

**Figure 16:**
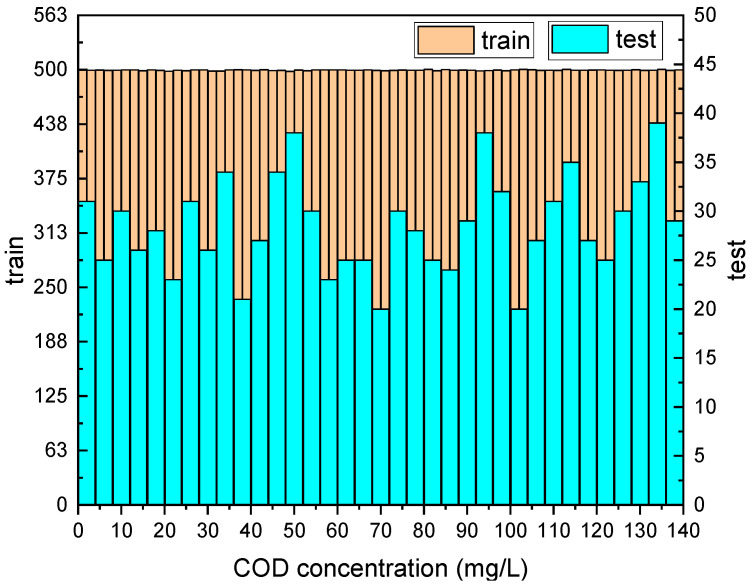
Distribution of spectral dataset after adding actual water sample spectral data.

To further validate the predictive effectiveness of this method for actual water body COD, actual water sample data were added to the training results based on the laboratory-configured amino acid solution. A total of 20 sampling points were selected from the main water systems in Hefei, Anhui Province, as shown in Figure 15. The sampling period was from March 2022 to March 2023, with two samplings per month. The absorption spectra and fluorescence spectra of the sampled water bodies were tested, with 10 spectral measurements repeated for each sampling point. The water body COD was tested using Lovibond Multidirect and Lovibond RD125, and the test results were taken as the label values. The specific names of the sampling points and the COD label values obtained after each sampling are shown in Table 6. In total, there were 4800 absorption spectra and fluorescence spectra data for the actual water samples. These actual water samples were combined with the laboratory water sample spectra for training, as shown in the distribution of the spectral dataset in Figure 16.

**Figure 17:**
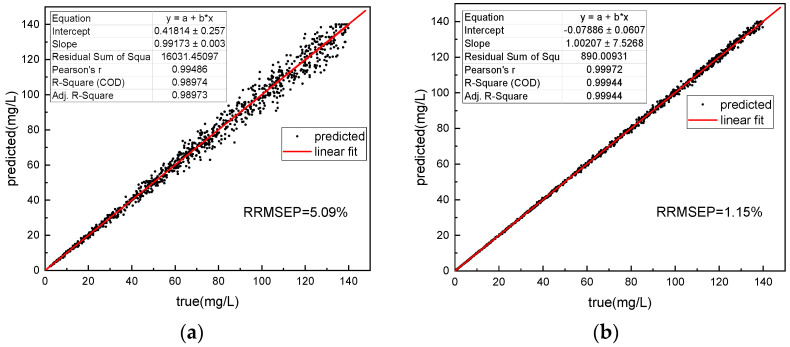
Comparison of COD prediction results between 1D-CNN method for absorption spectra (**a**) and fusion CNN for absorption–fluorescence spectra (**b**) in actual water samples.

The training results of the network are shown in Figure 17. The RRMSEP (Relative Root Mean Square Error of Prediction) for COD prediction using the single absorption spectral neural network method in actual water samples is as high as 5.09%. This is because in actual water samples with high COD concentrations, the presence of abundant organic compounds leads to significant overlap of absorption peaks in the absorption spectra. The information provided by a single absorption spectral data source is insufficient to accurately invert COD. In contrast, the fusion spectral neural network method effectively combines the fluorescence spectral information and absorption spectral information through the supervised learning of fluorescence spectra. Therefore, the COD retrieval is more accurate, and the RRMSEP for COD prediction is reduced to 1.15%.

## 4. Discussion

Aiming to solve the issue of inaccurate COD retrieval of amino acid mixed solution using a single spectral source, a COD retrieval algorithm based on the absorption–fluorescence spectrum feature fusion, called Fusion CNN, was designed to improve the accuracy of COD retrieval for amino acid mixed solutions. The absorption spectrum feature matrix was extracted through eight convolutional layers, and the fluorescence spectrum feature matrix was extracted through 2D Gabor with 35 scales/orientations. After dimensionality reduction using the Isomap method, the absorption–fluorescence spectrum feature matrix was fused through the fully connected layer, and the COD value retrieval was completed through a linear function. The fusion neural network algorithm model based on absorption–fluorescence spectral feature fusion offers advantages for COD retrieval. It extracts absorption spectrum features using 1D-CNN and reduces its dimensionality to match the feature matrix dimension of the 3D fluorescence spectrum, which is extracted using 2D Gabor transform to retain its two-dimensional spatial information and spectral information. The fusion of the one-dimensional feature vector and the two-dimensional feature matrix using feature concatenation leads to a more comprehensive description of the sample’s features and improves the accuracy of COD retrieval. Fully connected layers for feature extraction and dimensionality reduction comprehensively consider the spatial and spectral information of the absorption spectrum and 3D fluorescence spectrum features, reducing interference factors in the sample and improving retrieval accuracy. 

Further network training was conducted on the main water systems within Hefei City, Anhui Province. The training results showed that the fusion network outperformed the absorption spectrum CNN network in measuring COD accuracy in actual water bodies, with the RRMSEP improving from 5.09% to 1.15%.

## Figures and Tables

**Table 1:** COD test result of selected three types of amino acid organic compounds using the method of potassium dichromate.

Category	Name	Relative Molecular Mass	Measured Conversion Coefficient of COD	Concentration
Single-ring Structure	Tyrosine C_9_H_11_NO_3_ 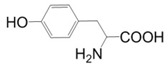	181.189	1.31	5 mg/L, 10 mg/L, 15 mg/L
	Phenylalanine C_9_H_11_NO_2_ 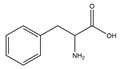	165.19	1.48	5 mg/L, 10 mg/L, 15 mg/L
Two-ring Structure	Tryptophan C_11_H_12_N_2_O_2_ 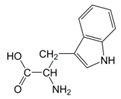	204.32	1.42	2 mg/L, 4 mg/L, 6 mg/L

**Table 2:** Absorption spectral dataset of three different types of mixed amino acid organic compounds.

Group	COD Value (mg/L)	Number of Absorption Spectra Groups after Data Augmentation
Tyrosine 5 mg/L + Tryptophan 2 mg/L	9.34	934
Tyrosine 10 mg/L + Tryptophan 4 mg/L	18.78	1878
Tyrosine 15 mg/L + Tryptophan 6 mg/L	28.17	2817
Tryptophan 2 mg/L + Phenylalanine 20 mg/L	29.60	2960
Tyrosine 5 mg/L + Phenylalanine 20 mg/L	36.15	3615
Tryptophan 4 mg/L + Phenylalanine 40 mg/L	64.88	6488
Tyrosine 10 mg/L + Phenylalanine 40 mg/L	72.30	7230
Tryptophan 6 mg/L + Phenylalanine 80 mg/L	126.92	12,692
Tyrosine 15 mg/L + Phenylalanine 80 mg/L	136.05	13,605
Tyrosine 15 mg/L + Phenylalanine 80 mg/L + Tryptophan 6 mg/L	146.57	14,657

**Table 3:** Comparison of different dimensionality reduction algorithms.

Dimensionality Reduction Algorithm	Run Time (Second)	$\mathrm{Average}\;\mathrm{Coefficient}\;\mathrm{of}\;\mathrm{Variation}\;(\bar{C_{v}})$
PCA	2.468	0.925
FastICA	1.709	1.232
Isomap	0.802	1.402
LLE	1.298	0.946
GRP	0.232	24.961~0.583

**Table 4:** Architecture Parameters of Fusion CNN.

Layer	Description	Dimension
Absorption Spectra Input Layer	Processes the raw absorption spectra data	2559
Fluorescence Spectra Input Layer	Processes the raw fluorescence spectra data	2217 × 2217
Fluorescence Spectra Feature Extraction Layer	2D Gabor transform and dimensionality reduction algorithm	5 × 128
16-Channel Dual-Channel 1D Convolutional Layer × 2	12 times of 3 × 1 1D convolutional operations, 2 max-pooling layers	639 × 16
32-Channel Dual-Channel 1D Convolutional Layer × 2	12 times of 3 × 1 1D convolutional operations, 2 max-pooling layers	159 × 32
64-Channel Dual-Channel 1D Convolutional Layer × 2	12 times of 3 × 1 1D convolutional operations, 2 max-pooling layers	39 × 64
128-Channel Dual-Channel 1D Convolutional Layer × 2	12 times of 3 × 1 1D convolutional operations, 2 max-pooling layers	19 × 128
Feature Concatenation Layer	Concatenates the feature matrices of fluorescence and absorption spectra	24 × 128
Fully Connected Layer	Feature fusion using a flatten layer and dense layer	1
Total Parameters	733,729	

**Table 5:** Training Parameter Comparison.

Algorithm	Training Parameter Count
Single-Channel Convolutional Neural Network	Approximately 5,500,000
Temporal Convolutional Neural Network (1D-TCN)	Approximately 1,600,000
Recursive Convolutional Neural Network (RCNN)	Approximately 5,000,000
Fractal-Structured Fusion CNN	733,729

**Table 6:** Details of sampling points and corresponding COD label values.

**Spot**	S1	S2	S3	S4	S5	S6	S7	S8	S9	S10
**COD (mg/L)**	30.3	29.8	21.2	17.1	36.0	44.1	25.0	54.1	38.2	33.5
**Spot Name**	Swan Lake Park	Swan Lake Park	Swan Lake Park	Swan Lake Park	Shiwuli River	Residential Area	Shiwuli River	Wanghu Park	Wanghu Park	Wanghu Park
**Spot**	S11	S12	S13	S14	S15	S16	S17	S18	S19	S20
**COD (mg/L)**	43.6	103.2	36.6	33.9	26.7	34.7	33.4	23.8	26.8	5.2
**Spot Name**	Shiwuli River	Fish Farm	Chaohu Lake	Nanfei River	Nanfei River	Hefei Harbor	Nanfei River	Nanfei River	Nanfei River	Dongpu reservoir

## Data Availability

The data that support the findings of this study are available from the corresponding author upon reasonable request.
